# The Veterans Affairs Precision Oncology Data Repository, a Clinical, Genomic, and Imaging Research Database

**DOI:** 10.1016/j.patter.2020.100083

**Published:** 2020-08-17

**Authors:** Danne C. Elbers, Nathanael R. Fillmore, Feng-Chi Sung, Spyridon S. Ganas, Andrew Prokhorenkov, Christopher Meyer, Robert B. Hall, Samuel J. Ajjarapu, Daniel C. Chen, Frank Meng, Robert L. Grossman, Mary T. Brophy, Nhan V. Do

**Affiliations:** 1 VA Cooperative Studies Program, VA Boston Healthcare System (151MAV), 150 S. Huntington Ave, Jamaica Plain, MA 02130, USA; 2 University of Vermont, Complex Systems Center, Burlington, VT 05405, USA; 3 Harvard Medical School, Boston, MA 02115, USA; 4 Dana-Farber Cancer Institute, Boston, MA 02215, USA; 5 University of Chicago, Center for Data Intensive Science, Chicago, IL 60615, USA; 6 Boston University School of Medicine, Boston, MA 02118, USA

**Keywords:** precision oncology, data commons, data sharing, translational research, healthcare, de-identification, secondary use of clinical data, data integration, genomic data, outcomes research

## Abstract

The Veterans Affairs Precision Oncology Data Repository (VA-PODR) is a large, nationwide repository of de-identified data on patients diagnosed with cancer at the Department of Veterans Affairs (VA). Data include longitudinal clinical data from the VA's nationwide electronic health record system and the VA Central Cancer Registry, targeted tumor sequencing data, and medical imaging data including computed tomography (CT) scans and pathology slides. A subset of the repository is available at the Genomic Data Commons (GDC) and The Cancer Imaging Archive (TCIA), and the full repository is available through the Veterans Precision Oncology Data Commons (VPODC). By releasing this de-identified dataset, we aim to advance Veterans' health care through enabling translational research on the Veteran population by a wide variety of researchers.

## Highlights

• Presents a large-scale repository on cancer patients with EHR, imaging, and genomic data
• Describes a method for de-identification of different types of health data
• Enables researchers to use data science tools to apply to the data

## The Bigger Picture

Accelerating the speed of innovation and discoveries in health care requires the liberation of real-world data from their silos. One of the greatest challenges in the application of artificial intelligence and machine learning to health care is the validation of new algorithms beyond where they were created. We present the Veterans Affairs Precision Oncology Data Repository (VA-PODR), a large-scale repository of de-identified data on patients diagnosed with cancer at the Department of Veterans Affairs (VA). VA-PODR includes longitudinal clinical, genomic, and imaging data originating from the VA's electronic health record system, the VA Central Cancer Registry, and other sources. VA-PODR enables researchers around the world to validate their algorithms and advance cancer research and health care in general. In addition, VA-PODR enhances Veterans' health care by facilitating development of algorithms that are well tuned to the Veteran population and ready for deployment inside the VA.

## Teaser

The Veterans Affairs Precision Oncology Data Repository (VA-PODR) is a large, nationwide repository of real-world de-identified data on patients diagnosed with cancer at the Department of Veteran's Affairs. The repository contains longitudinal clinical data from the VA's nationwide electronic health record system and the VA Central Cancer Registry, targeted tumor sequencing data, and medical imaging data. By releasing this dataset, we aim to advance Veterans' health care through enabling translational research on the Veteran population by a wide variety of researchers.

## Introduction

The Department of Veterans Affairs (VA) operates the largest integrated health care system in the United States and was an early adopter of electronic health record (EHR) systems.^1^ As a result, the VA has large longitudinal databases containing health records dating back as far as the 1980s, with comprehensive coverage starting in 1999.^2^ More recently, the VA launched the Precision Oncology Program (POP), initially in the New England region under leadership of our group at the Boston Cooperative Studies Program (CSP) Informatics Center,^3^^,^^4^ and subsequently as a national program led by the VA's National Oncology Program.^5^ Under POP, the VA has carried out targeted tumor sequencing on a national scale for Veterans diagnosed with cancer.

Although the VA has established successes in the care for cancer patients through evidence-based approaches, to accelerate this progress even further, it is critical to combine expertise from both inside and outside the VA. To that end, our group established the Research Precision Oncology Program (RePOP), which provides a mechanism for patients to consent to broad data sharing for research purposes. This, along with additional work to assure regulatory compliance, enables the VA to contribute data into the cancer data ecosystem as recommended by the Cancer Moonshot Blue Ribbon Panel.^6^^,^^7^ For example, consent obtained under RePOP has facilitated the VA's participation in the Cancer Moonshot's Applied Proteogenomics OrganizationaL Learning and Outcomes (APOLLO) network.^8^

Due to these efforts, we are now able to share VA data as a national resource to investigators inside and outside the VA. Specifically, in this paper, we introduce the VA Precision Oncology Data Repository (VA-PODR) and describe its availability outside the VA. VA-PODR consolidates de-identified VA clinical, genomic, and imaging data needed for research in precision oncology in a large, nationwide repository. The data consist of longitudinal clinical data from the VA's integrated EHR system and the VA Central Cancer Registry, targeted tumor sequencing data, and medical imaging data including computed tomography (CT) scans and pathology slides.

In previous work, we described the VPODC, a data-sharing and computational platform where VA-PODR is available to researchers outside the VA.^9^ Here, in contrast, we describe the VA-PODR data repository itself. The difference between the VPODC and VA-PODR is that the VPODC is a specific platform through which the VA-PODR dataset is shared and collaborative analysis can take place. However, the VPODC is not the only place where VA-PODR is available. Parts of VA-PODR are also shared in the Genomic Data Commons (GDC)^10^ and The Cancer Imaging Archive (TCIA),^11^ as well as internally at the VA. And, in the future, it is our intention that VA-PODR will be made available elsewhere as well.

VA-PODR represents a unique resource, for several reasons. First, to our knowledge, VA-PODR is the first large-scale repository of VA health data that has ever been made available to researchers outside the VA. Second, VA-PODR is one of only a handful of large-scale databases with real-world EHR data to be made available to the research community outside the institutions where the data originated, similar to the widely used MIMIC Critical Care Database,^12^ but in a different medical domain. Third, VA-PODR combines clinical, genomic, and imaging data, enabling multimodal analysis that is not possible with only one source, similar in some respects to The Cancer Genome Atlas (TCGA),^13^ but with substantially richer clinical data.

In addition to VA-PODR's use for observational research in precision oncology, we envision VA-PODR facilitating big data research in health care in general, such as for the development and validation of analytical models and other tools using large EHR, genomic, and imaging data, and also being used for educational purposes. Thus, our contribution will benefit both Veterans and the broader community by providing accessible data to accelerate improvement in health care.

## Results and Discussion

### Methods

#### Repository Development

**Figure 1:**
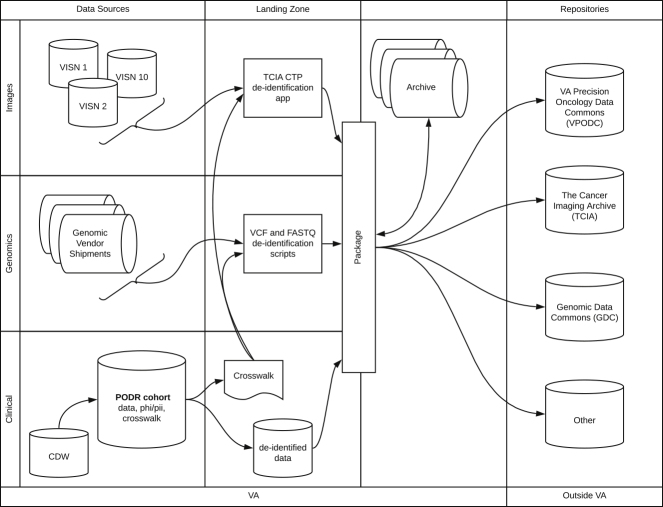
Overview of the VA-PODR Dataflow Data are pulled from several sources within the VA, aligned and de-identified in the landing zone, and subsequently submitted to collaborating repositories.

VA-PODR consists of (1) de-identified longitudinal clinical data from the VA's integrated EHR system and the VA Central Cancer Registry, (2) targeted tumor sequencing data from POP, and (3) medical imaging data including CT scans and pathology slides. Data from multiple sources is pulled to a central location, and records are matched using internal patient identifiers (Figure 1).

Clinical data include 10 domains, 38 tables, and 647 columns, and approximately 1 billion rows of data from the VA's EHR system, with detailed information on demographics, survival, laboratory test results, orders, medications, surgeries, and visits, including associated ICD and procedure codes, and more. This information is available in the VA Corporate Data Warehouse's native data model as well as in the VA's implementation of the Observational Medical Outcomes Partnership (OMOP) Common Data Model (CDM). Our intention is for VA-PODR to include all information from the EHR that is relevant for research. In addition, extensive curated data on cancer diagnosis, treatment, and outcomes is included from the VA Central Cancer Registry (VACCR),^14^ which collates data on cancer cases that have been annotated by VA cancer registrars throughout the nation.

Genomic data include data generated under both the VA New England Healthcare System Precision Oncology Program^3^^,^^4^ and the VA National Precision Oncology Program.^5^ These data consist of targeted tumor sequencing data, including both raw sequencing files and somatic variant calls.

Medical imaging data are extracted from two distinct sources. CT scans taken at or near diagnosis are pulled from various VA's medical centers' Picture Archiving and Communication Systems. In addition, images of pathology slides from sequenced tumor biopsies are included; these were produced by the vendors carrying out the targeted tumor sequencing described above, or are histopathology slides digitized at local pathology departments.

#### De-identification

##### General Strategy

Before release, all data are de-identified in accordance with both the United States Health Insurance Portability and Accountability Act (HIPAA)^15^ and internal VA requirements, including VHA Handbook 1200.12 guidelines.^16^ Specifically, all data elements covered under HIPAA as identifiers are removed, as well as other possible sensitive information, identified as such by subject matter experts (SMEs); see details below. After de-identification using the procedures below, all data are reviewed and approved by a VA Privacy Officer before release.

In all data types, dates are obfuscated by calculating days to an arbitrary patient-specific anchor date. This procedure preserves the ordering of dates within each patient's timeline. No patient data are included for which the timestamp indicates that it was collected at the time a patient is 90 years of age or older.

##### Clinical Data

**Figure 2:**
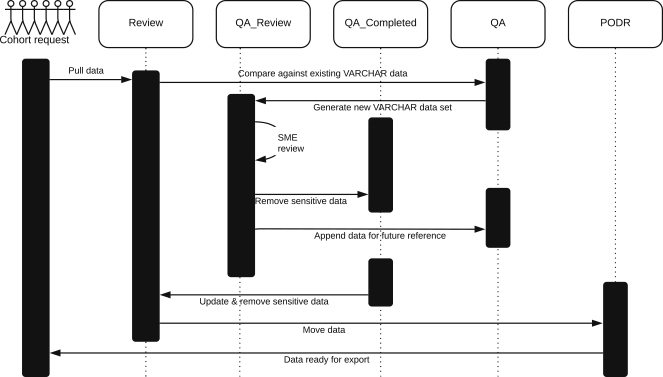
Review Process to Exclude Sensitive and Identifiable Data Once a cohort is requested, new data are pulled and unique values are compared with data values previously evaluated. New data values are evaluated by SMEs and either white- or blacklisted. The new dataset is filtered by the white-listed dataset, de-identified, and shared.

As briefly mentioned above, the clinical data are de-identified through a manual review for identifiers under HIPAA, sensitive information, and/or other PHI/PII by SMEs. Specifically, each column of data is assessed for inclusion or exclusion based on whether it primarily contains this type of information, which can occur in categorized fields or free text, or not. Columns that primarily contain sensitive information are excluded. In addition, for columns that are included, each distinct data value is evaluated for sensitivity by an SME and either excluded or added to a white list. Examples of sensitive data are medication discontinued dates, serial codes, and references to physicians and locations. As new data are added, they are checked against the white list and flagged for manual review if not present (Figure 2). Thus, SMEs are involved in two steps: first in assessing each new column and deciding if the column in its entirety contains sensitive and/or HIPAA information and second in reviewing new and distinct varchar values and deciding if this specific value should be white- or blacklisted while the column itself is part of PODR.

##### Genomic Data

Genomic data are de-identified by removing any HIPAA identifiers occurring in metadata associated with each record and certifying this de-identification methodology by an SME. Genomic data of tumors are not considered inherently identifiable under US regulations.^17^

##### Imaging Data

For imaging data, we ensure that both headers and image content do not include sensitive or identifying information. Sensitive data in Digital Imaging and Communications in Medicine (DICOM) headers are removed through a process similar to that used for clinical data. To deidentify image content, we use the Medical Imaging Resource Community's Clinical Trials Processor software.^18^

#### Technical Validation

Data have been validated three ways. Best practices have been used in developing code to pull and collate data, relying on standard internal VA identifiers to join data across domains where possible, and subsequently translating these to public identifiers. In addition, all data domains and values have been reviewed by a group of SMEs with expertise in medicine, imaging, and data analysis. Finally, all data have been reviewed and certified as de-identified by a VA Privacy Officer before release.

##### Risk of Re-identification

In sharing VA-PODR data, de-identification standards have been adopted, extended, and thoroughly vetted to protect VA patients. In order to further mitigate residual re-identification risk in the de-identified datasets, we have also implemented policy controls. Specifically, the larger set of de-identified data is available only after proof of institutional privacy policies, vetting of data transfer rules, and signing of a Data Use Agreement (DUA), in which the user agrees not to attempt re-identification. These measures are further detailed in the next section.

#### Regulatory Considerations

In accordance with VA regulations, VA data can be housed in a database, a single repository, or a data repository. A database is explicitly created in the course of conducting research, while a single repository can be used to archive/compile data from multiple protocols and/or investigators working on similar topics. VA-PODR is a data repository, meaning that it is a living document that details the sources and contents of archived research data and also describes all secondary use of data including where the data go, who uses them, for what purposes, over time. VA-PODR currently describes two cohorts, A and B, with their regulations detailed below. We anticipate other cohorts will be added to VA-PODR in the future, whether prospective or retrospective, observational or interventional, of which APOLLO is an example.

##### Cohort A Consent and HIPAA Authorization

For patients in cohort A, consent and HIPAA authorization was obtained to share data with partners inside and outside the VA. Inside the VA, data on patients in cohort A can be shared either identified or de-identified while coded or anonymized, depending on the investigator's protocol and the details of the DUA signed with PODR. Outside the VA, data on patients in cohort A can only be shared after they are de-identified and coded or anonymized and a DUA is signed with PODR. This DUA may allow data on patients in cohort A to be reshared. The signed DUA will state that re-identification is prohibited.

##### Cohort B Decedent and HIPAA Waived

Patients in cohort B need to be recorded as decedent in their medical record; identification of these patients is performed under an HIPAA waiver. Decedent data are not considered data on human subjects. Similar to cohort A, inside the VA, data on patients in cohort B can be shared either identified or de-identified while coded or anonymized, depending on the investigator's protocol and the details of the DUA signed with PODR. Outside the VA, data on patients in cohort B can only be shared after they are de-identified and coded or anonymized and under a DUA signed between PODR and a trusted partner with a standard operating procedure or institutional review board-approved protocol. Different from cohort A, these data cannot be reshared and must remain in the trusted partner's repository. The signed DUA will state that re-identification is prohibited.

##### Unanticipated Events

If an unanticipated event occurs with PODR data, the procedures in the most recent version of the PODR protocol and VA handbook 1200.05 are followed. The event is reported to the privacy officer, principal investigator, quality assurance manager, institutional review board, and R&D committee at the VA Boston Healthcare System. In addition, the event is entered into a national privacy database by the privacy officer, and a determination is made on the need for corrective action.

### Results

#### Patient Characteristics

In its current release, VA-PODR includes data on 113,154 Veterans diagnosed with cancer at the VA, including 1,115 patients who were enrolled in POP. Details on demographics, date of diagnosis, and cancer type are shown in Tables 1, 2, and 3. Patients in VA-PODR are predominantly older (26.7% ≥80 years, 32.8% 70–79 years, 33.7% 60–69 years, 6.4% 50–59 years, and only 0.4% <50 years) and male (98.8% male, 1.2% female). The cohort includes 18.1% African American and 68.3% white patients. Cancer types include prostate (58,323 patients), lung (56,836), bladder (3,640), skin (2,168), colon (2,043), and kidney (1,284) cancer, among others. Year of diagnosis ranges from 2005 to 2019, with 2.6% of diagnoses in 2004 or earlier.

**Table 1:** Demographic Characteristics of the VA-PODR Patient Population

Characteristic	VA-PODR (N = 113,154), n (%)
Age	
<50 years	391 (0.4)
50–59 years	7,215 (6.4)
60–69 years	38,170 (33.7)
70–79 years	37,151 (32.8)
≥80 years	30,227 (26.7)
Gender	
Male	111,811 (98.8)
Female	1,343 (1.2)
Race	
African American	20,531 (18.1)
American Indian	512 (0.5)
Asian	247 (0.2)
White	77,295 (68.3)
Native Hawaiian/Pacific Islander	668 (0.6)
Unknown	13,901 (12.3)
Ethnicity	
Hispanic or Latino	3,624 (3.2)
Not Hispanic or Latino	99,862 (88.3)
Unknown	9,668 (8.5)

Patients can report multiple races.

**Table 2:** Distribution of Cancer Types in the VA-PODR Patient Population as Reported by the VACCR

Cancer Type	VA-PODR (N = 113,154), n (%)
Prostate	58,323 (51.5)
Lung	56,836 (50.2)
Bladder	3,640 (3.2)
Skin	2,168 (1.9)
Colon	2,043 (1.8)
Kidney	1,284 (1.1)
Other	11,963 (10.5)

Patients can have multiple cancer types reported.

**Table 3:** Year of Diagnosis of Cancer in the VA-PODR Patient Population as Reported by the VACCR

Year of Diagnosis	VA-PODR (N = 113,154), n (%)
≤2004	2,986 (2.6)
2005	7,158 (6.3)
2006	6,658 (5.9)
2007	6,918 (6.1)
2008	6,004 (5.3)
2009	6,752 (6.0)
2010	12,517 (11.1)
2011	11,710 (10.3)
2012	10,742 (9.5)
2013	9,784 (8.6)
2014	9,203 (8.1)
2015	8,316 (7.3)
2016	6,698 (5.9)
2017	5,080 (4.5)
2018	2,336 (2.1)
2019	106 (0.1)
Unavailable	270 (0.2)

Efforts are underway to expand VA-PODR to include all lung cancer patients within the VA since 1999 (approximately 150,000 cases) and add at least 100,000 prostate cases. Ultimately, we intend all known cancer cases at the VA to be included in VA-PODR. We are also conducting iterative updates of images and genomic data as they become available, as well as expanding clinical data domains to, for example, include the inpatient and radiology domains. We are open to input from the research community on determining expansion priorities.

#### Classes of Data

Clinical data are stored in a relational database for the following domains: outpatient visits, inpatient medications, outpatient medications, all laboratory test results, all orders, surgery, and patient demographics. Clinical data also include cancer registry data and a derived table containing all ICD codes and timestamps associated with each patient across several domains (Table 4).

**Table 4:** Data Domains Available in VA-PODR

Domain Table Name	Description
BCMAMedicationLog	Inpatient medications
CPRSOrder	All orders for drugs, labs, etc.
Outpat_VDiagnosis, Outpat_Visit, Outpat_VPatientEd, Outpat_VProcedure, Outpat_VProcedureDiagnosis, Outpat_VSkinTest, Outpat_VSkinTestDiagnosis	Outpatient visit information
PatientLabChem	Laboratory test information
PatientMeansTest	Patient income information
Patients	Basic demographic and vital status information
RxOutpat	Pharmacy outpatient
SurgeryPRE, SurgeryINTRA, SurgeryPOST, SurgeryProcedureDiagnosisCode	Surgery data
ICDCode	All ICD codes with timestamp
Oncology_Primary	VA Central Cancer Registry Data
OMOP	The Observational Medical Outcomes Partnership (OMOP) Common Data Model (CDM) V5

Genomic data are stored in individual files, linked to clinical data by filename identifiers. Genomic data include three levels of detail: raw sequencing data in FASTQ format, somatic mutation data stored in VCF format, and information on actionable mutations based on curation by molecular diagnostic vendors. Two vendors were used: Personalis, which used the ACE Cancer Plus panel, and PGDx, which used the Cancer Select 203 panel. Information on which vendor was used for each patient sample is available in the metadata.

Imaging data are stored as DICOM stacks in Orthanc,^19^ an open source PAC server, and are linked to the clinical data by patient identifiers. A set of the metadata extracted from the DICOM tags is available in table format for direct querying.

#### Clinical Data Domains

VA-PODR contains a broad range of longitudinal clinical data both from the VA's EHR system and from the VACCR. EHR data in VA-PODR include information on patient demographics, comorbidities, procedures, medications, laboratory test results, medical orders, survival, as well as detailed administrative information arising from inpatient and outpatient visits to the VA. In addition, VACCR data in VA-PODR include extensive manually annotated information on cancer cases and outcomes.

#### Repository Access and Locations

VA-PODR at present consists of two different cohorts with different access policies. Cohort A includes patients who have consented to broad data sharing with parties external to the VA under the RePOP protocol. Cohort B includes patients who are deceased and are not considered human subject research, but by internal policy is subject to additional restrictions compared with cohort A. In the future, additional cohorts may be added.

A subset of VA-PODR data from patients in cohort A is available at the Genomic Data Commons^10^^,^^20^ and TCIA,^11^^,^^21^ and our intention is that relevant data elements from all of cohort A will be available at these locations in the future. All VA-PODR data for cohort A are available at the Veterans Precision Oncology Data Commons (VPODC)^22^ to approved users who provide proof of institutional privacy policies and data transfer rules to the data steward. Cohort B is also accessible at the VPODC through the data steward and with the approval of the data owner (VA). Like the GDC, the VPODC is a platform for data sharing and analysis in the Gen3 commons framework developed by the University of Chicago.^20^

To request access to VA-PODR via VPODC, users should send an access request email to the contact listed at https://vpodc.org. The initial email should include a description of the research topic, the cohort to which access is requested, and institutional details. Access requests will be reviewed and prioritized by an allocations committee. Before data access is granted, all users will be required to complete training on privacy and information security. In addition, users will be required to sign the applicable and most recent version of the DUA.

#### FAIR Principles

VA-PODR aligns with the FAIR principles^23^ that data should be findable, accessible, interoperable, and re-usable. A full data dictionary is included in VA-PODR and available at the VPODC. In the GDC and TCIA, the data follow the data structures mandated and documented by those frameworks, creating a findable and rich metadata structure with unique and persistent identifiers. The VPODC, GDC, and TCIA follow interoperable standards for authentication, access, and retrieval. The subset of VA-PODR data that follows the OMOP data structure allows for interoperability with other health care data repositories, and the subset of data available at GDC and TCIA follows those systems' standards. Since data will be housed long-term at these sites, it will remain findable and re-usable. Thus, we believe that the VA-PODR satisfies the conditions of being FAIR.

#### Use Cases

In addition to VA-PODR's primary use to enable research in precision oncology, the dataset has substantial use for several other purposes of broad interest, including (1) methods research in analysis using EHR data, (2) educational and training purposes, and (3) development of clinical informatics tools. VA-PODR is particularly valuable for these additional purposes, because there are currently few large EHR datasets readily available to researchers or students who are not affiliated with an institution that has an EHR data warehouse.

To date, VA-PODR data have been used in the Department of Commerce's The Opportunity Project (TOP) Health Artificial Intelligence initiative.^24^^,^^25^ In addition, VA-PODR data have been used to carry out external validation and calibration of a prognostic model for mortality among patients with non-small cell lung cancer.^26^^,^^27^ In addition, research projects on lung and prostate cancer using VA-PODR data and the VPODC platform are underway.

### Conclusion

We have described VA-PODR, a large, nationwide repository of de-identified data on patients diagnosed with cancer at the VA. This repository contains longitudinal clinical data from the VA's nationwide EHR system and the VACCR, targeted tumor sequencing data, and medical imaging. A subset of the repository is available at the GDC and TCIA, and the full repository is available through the VPODC. By making these data available, VA-PODR enables multiple uses of benefit to both Veterans and the broader community.

## Experimental Procedures

### Resource Availability

#### Lead Contact

Danne Elbers is the lead contact of this study and can be reached by email: danne.elbers@va.gov.

#### Materials Availability

This study did not generate new materials.

#### Data and Code Availability

VA-PODR is available at https://vpodc.org, and subsets of the data are available at https://portal.gdc.cancer.gov/projects/VAREPOP-APOLLO and https://wiki.cancerimagingarchive.net/display/Public/APOLLO-1-VA.The published article reports on all data generated to date (July 25, 2020), and it is anticipated that the data will be expanded. There are restrictions to the availability of data due to privacy considerations, as described above.
